# Diverse Functionalities of Vertically Stacked Graphene/Single layer n-MoS_2_/SiO_2_/p-GaN Heterostructures

**DOI:** 10.1038/s41598-017-09998-1

**Published:** 2017-08-30

**Authors:** Packiyaraj Perumal, Chelladurai Karuppiah, Wei-Cheng Liao, Yi-Rou Liou, Yu-Ming Liao, Yang-Fang Chen

**Affiliations:** 1 Department of Physics, National Taiwan University, Taipei, 106 Taiwan; 2 Nano Science and Technology Program, Taiwan International Graduate Program, Academia Sinica and National Taiwan University, Taipei, 106 Taiwan; 3 Center for Emerging Material and Advanced Devices, National Taiwan University, Taipei, 106 Taiwan; 4 Department of Chemistry, National Taiwan University, Taipei, 106 Taiwan

## Abstract

Integrating different dimentional materials on vertically stacked p-n hetero-junctions have facinated a considerable scrunity and can open up excellent feasibility with various functionalities in opto-electronic devices. Here, we demonstrate that vertically stacked p-GaN/SiO_2_/n-MoS_2_/Graphene heterostructures enable to exhibit prominent dual opto-electronic characteristics, including efficient photo-detection and light emission, which represents the emergence of a new class of devices. The photoresponsivity was found to achieve as high as ~10.4 AW^−1^ and the detectivity and external quantum efficiency were estimated to be 1.1 × 10^10^ Jones and ~30%, respectively. These values are superier than most reported hererojunction devices. In addition, this device exhibits as a self-powered photodetector, showing a high responsivity and fast response speed. Moreover, the device demonstrates the light emission with low turn-on voltage (~1.0 V) which can be realized by electron injection from graphene electrode and holes from GaN film into monolayer MoS_2_ layer. These results indicate that with a suitable choice of band alignment, the vertical stacking of materials with different dimentionalities could be significant potential for integration of highly efficient heterostructures and open up feasible pathways towards integrated nanoscale multi-functional optoelectronic devices for a variety of applications.

## Introduction

Atomically thin two-dimensional (2D) layered semiconductors, such as transition metal dichalcogenides (TMDs) has attracted tremendous interest in nanoscale semiconductor devices owing to their attractive scrunity both in academic and industrial interest^1–7^. Even though, the applications for graphene in optoelectronic devices is still limited by its zero band gap, 2D TMDs with their variety of band gaps have fascinating in various advanced devices such as field effect transistors (FET)^8^, photo-detectors^9, 10^, photovoltaics^11^, and light emitting diodes (LEDs)^12, 13^. In particular, the single layer (1-L) MoS_2_, another graphene analogue with a direct optical bandgap of ~1.80 eV, has been scrutinised extensively due to its high absorption coefficient, strong photoluminescence (PL) and efficient electron-hole pair generation under illumination of light. Because of these remarkable characteristic of 1-L MoS_2_, a series of functional devices, including FETs, memories, photo-diodes, LEDs, and sensors have been succesfully demonstrated^14–16^. For example, 1-L MoS_2_ exhibits mobilities >60 cm^2^ V^−1^S^−1^, a photogain up to 24, a detectivity ~10^10^ Jones, and a response time of ~40 μs. Additionally, top-gated 1-L MoS_2_ FET has been demonstrated to display a high carrier mobility ~200 cm^2^ V^−1^ s^−1^ and on/off ratio of ~10^8^. These results delegate that 1-L MoS_2_ can be a superior candidate material for both eletronics and optical devices^17^. To further enhance the potential opto-electronic applications, atomically thin 2D TMDs based heterostructures have emanated, and attained a lot of scrutiny recently^18^. Especially, van der Waals (vdW) heterostructures with different types of TMDs have been developed for encouraging well defined devices^19^. As a consequence, laterally or vertically aligned heterostructures based on 1-L MoS_2_, such as MoS_2_/graphene, MoS_2_/WS_2_ or WSe_2_ and MoS_2_/CNT, have also been explored to realize various functionalities such as excellent photodetection, LEDs, sensors and memory devices^13, 20–22^. However, several restrictions to produce vdW heterostructures of 2D TMDs demand complicated self-profelled arrangement between two different layers for the construction of large areas with high quality electrical contacts, which still remains as a great challenge^23^. Considering these issues, if 3D substrates is used to integrate with 2D materials, to fabricate efficient 3D-2D heterostructures with superior properties^24, 25^, which are accordant with the functions of combined complementary metal oxide semiconductor (CMOS) technology. It will be able to generate novel devices with excellent performance to have a great potential for practical applications.

The conventional 3D semiconductors, such as Si, Ge, SiC, GaAs, GaN, SnO and ZnO are the foremost elementary building blocks of modern solid-state electronics to create excellent heterojunctions^26–29^. The significant advantage of easy to transfer 2D TMDs grown by CVD on a 3D semiconductor substrate emerges as an outstanding prospective to produce a large area vertical heterojunction diode with excellent device performance^26^. Although a few studies on integrating 2D TMDs with 3D semiconductors have been developed very recently, they mainly focused on the electrical performance of the heterojunction. For example, ZnO/graphene, Si/graphene and GaN/graphene^30^ have been identified to exhibit good Schottky junctions. Among all, GaN has a direct wide-bandgap of 3.4 eV and p-type doping in GaN has been proven to be feasible. The integration of 2D layered TMDs with III-nitrides could lead to the flexibility of new and exciting device engineering^31^. Very recently, several works have reported GaN-based optoelectronic devices with excellent performances such as LEDs and solar cells based on graphene/p-GaN^32^. However, a thorough study of heterostructure consists of vertically stacked graphene and MoS_2_ on GaN has not yet been reported, although 3D-2D heterojunctions are of specific interest as they permit for rapid development of these varieties of devices at industrial applications.

In this work, we report the novel fabrication and unambiguosly demonstrate multifunctionalities of p-GaN/SiO_2_/1-L n-MoS_2_/graphene based vertically stacked p-n heterostructures. The 1-L MoS_2_ and graphene were assembled vertically by a wet chemical transfer technique on p-GaN/Sapphire substrate. This intriguing device structure was confirmed by confocal photoluminescence and Raman spectroscopies. From the electrical measurements, the device exhibits a clear current-rectifying characteristics. The diode-like behavior of these heterostructures was ascribed to the intrinsic built-in electric field generated at the interface. Under illumination of 633 nm laser, this p–n heterostructure exhibits a strong photoresponse of about ~10.4 AW^−1^ with an external quantum efficiency (EQE) of 30%. The high photoresponsivity can be attributed to the strong electric field at the interface and also the short carrier diffusion path. Significantly, the device can act as a self-powered photodetector operated at zero bias. In addition, the observed light emission from this p-n junction diode exhibits in the range of ~650–700 nm under low turn-on forward bias condition. Our results can be understood well based on the mechanism of the carrier transfer between these unique monolayer heterostructures in terms of the suitable band alignment. This study reveals the inherent nature of vertical stacking of monolayer p-n heterojunctions derived from materials with different dimentionalities for future large area and multifunctional integrated high performance optoelectronic devices, which should be very useful and timely.

## Results and Discussion

**Figure 1:**
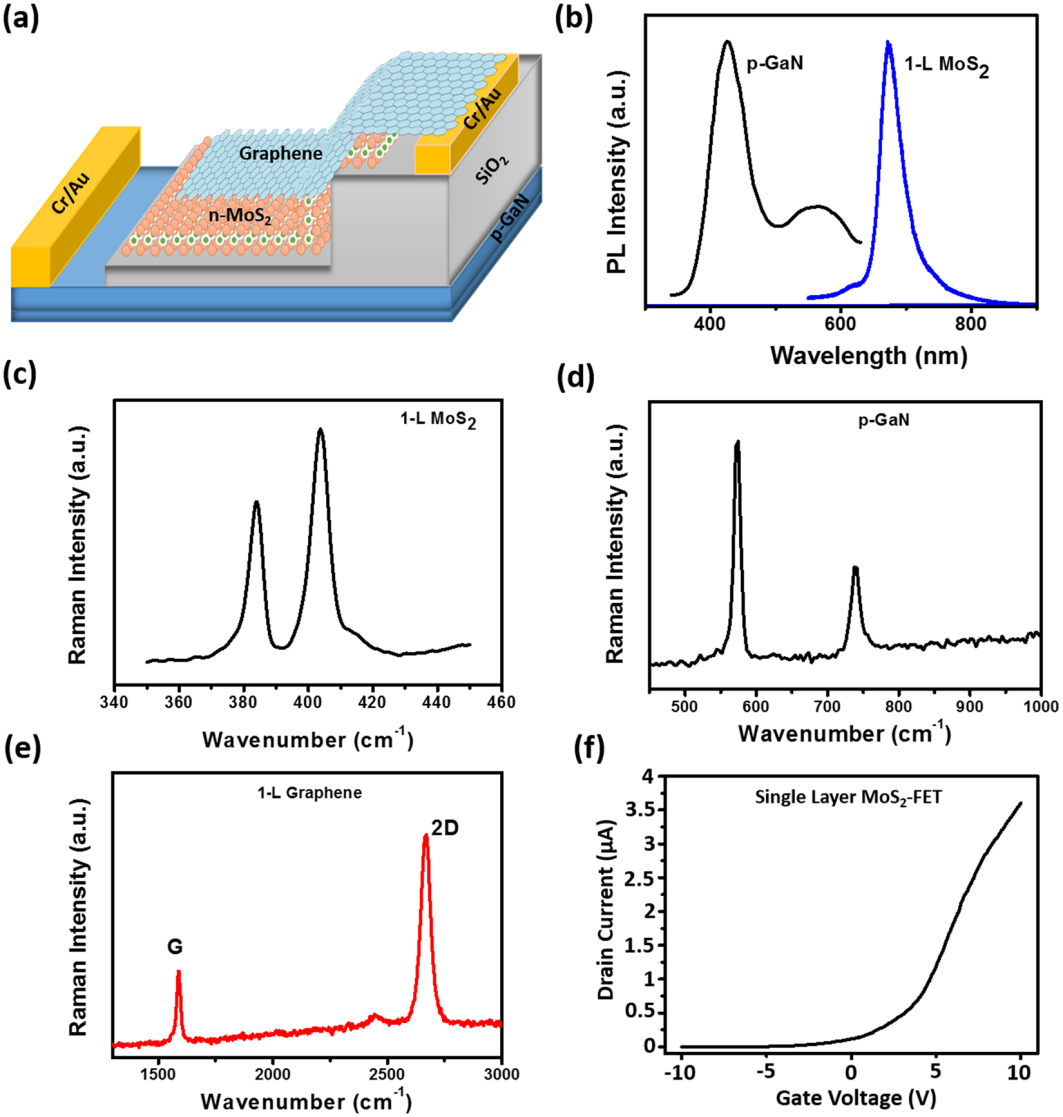
(**a**) Schematic device structure consists of p-GaN/SiO_2_/n-MoS_2_/graphene heterostructure. (**b**) Confocal photoluminescence emission spectra of p-GaN and single layer MoS_2_. Confocal Raman spectra for (**c**) Single layer MoS_2_, (**d**) p-GaN and (**e**) graphene. (**f**) *I*_ds_-*V*_g_ characteristic curve for single layer MoS_2_-FET measured at *V*_g_ from −10 to 10 V under dark.

Figure 1a shows the schematic illustration of the heterojunction device structure. The heterostructure consists of p-GaN/SiO_2_/1-L n-MoS_2_/Graphene, in which the layered materials are aligned vertically to the substrate. Highly doped p-GaN, 1-L MoS_2_ and graphene were grown by metal organic chemical vapor deposition (MOCVD) on sapphire, CVD on Si/SiO_2_ and Cu substrate, respectively. The 2D layered materials such as 1-L MoS_2_ and graphene were organized on p-GaN/sapphire by using wet chemical transfer technique. Before transfering layered materials, an insulating 10 nm thickness of SiO_2_ film was deposited on the surface of p-GaN to serve as carrier blocking layer and to avoid the rapid carrier leakage across the single MoS_2_ layer. Next, 1-L MoS_2_ was transferred onto SiO_2_/p-GaN/sapphire to realize a heterojunction of p–n diode. Two Cr/Au metal electrodes of 5 nm/70 nm were deposited according to the schematic diagram shown in Fig. 1a to act as top and bottom electrodes, respectively. To improve the carrier injection, graphere was transfered on top of 1-L MoS_2_. The detailed fabrication process is discussed in the experimental section.

The photoluminescence (PL) spectra of p-GaN and 1-L MoS_2_ are organized together for comparision as shown in Fig. 1b. The strong peak at ~415 nm of p-GaN indicates the near bandgap emission, which confirms that as-grown GaN has a high crystallinity and good p-type characteristics^33^. The weak and broad peak at ~550 nm is caused by the dislocation of atoms or native point defects emission^34^. The strong PL emission spectra of 1-L MoS_2_ noticed at ~680 nm supports the direct bandgap of ~1.8 eV attributed to the near band gap transition. To furthur evaluate the thickness, optical characteristics and vertical formation of 1-L MoS_2_, p-GaN and graphene, the confocal Raman spectra measurements were carried out and shown in Fig. 1c–e. In Fig. 1c, the MoS_2_ film has strong E^1^_2g_ and A_1g_ Raman peaks, which are perceived at 385 and 403 cm^−1^, respectively. The seperation between these two peaks is 18 cm^−1^, indicating that the obtained MoS_2_ is a 1-L with smooth surface. Figure 1d displays the E_2_^high^ and A_1_^LO^ characteristic peaks for p-GaN at 570 and 735 cm^−1^, respectively. As can be seen in Fig. 1e, the intensity ratio between G and 2D peak reveals that graphene has a high quality single layer or bilayer. Additionally, to verify each layer is stacked vertically to form p-GaN/SiO_2_/n-MoS_2_/graphene heterostructures as depicted in Fig. 1a, the spectrum employed by the confocal Raman spectra is shown in Figure S1 in the Supporting Information. These results indicate that graphene and MoS_2_ mono layers are well stacked vertically on top of GaN/Sapphire substrate because the spectrum does signify the appearance of each corresponding layer^35–39^. In addition, to confirm the n-type conduction feature, the as-prepared 1-L MoS_2_ has been used to fabricate back gated-FETs based on MoS_2_/SiO_2_/Si for perceiving the output transport behaviour, as displayed in Fig. 1f. From the drain current versus gate voltage (I_D_–V_G_) characteristics, we can see that the I_D_ increases as V_G_ is swept from −10 to +10 V, indicating that the electron conduction is dominant in the 1-L MoS_2_ FET device.

**Figure 2:**
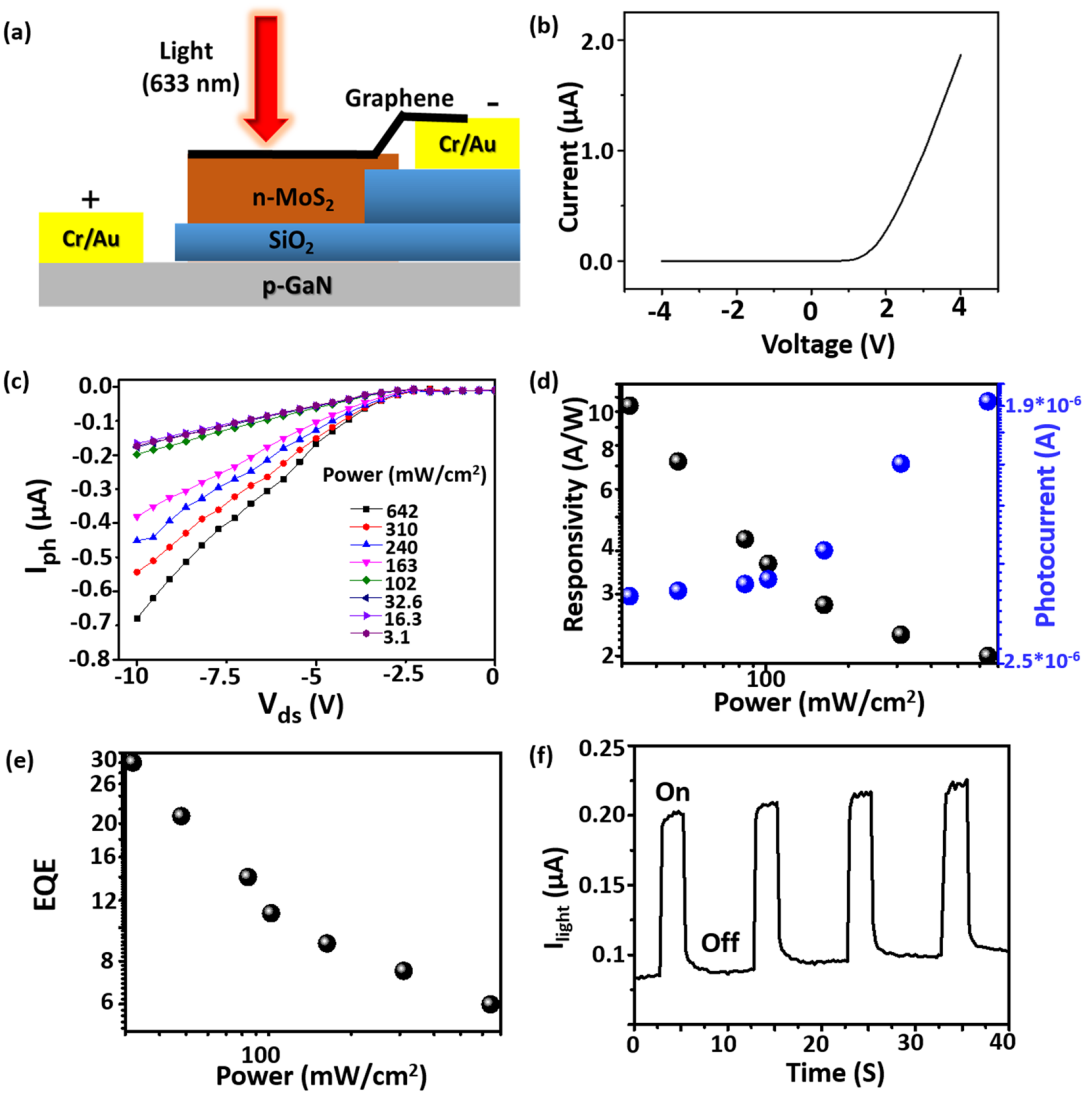
(**a**) Schematic device structure for the photodetection of p-GaN/SiO_2_/n-MoS_2_/graphene heterostructure photo-detector. (**b**) *I*–*V* characteristics of p-GaN/SiO_2_/n-MoS_2_/graphene photodetector under dark. (**c**) The photoresponse spectra of a p-GaN/SiO_2_/n-MoS_2_/graphene photodetector measured under different illuminated power intensity (642, 310, 210, 163, 102, 32.6, 16.3, 3.1 mW/cm^2^). (**d**) The plot of photoresponsivity and photocurrent versus laser power intensity. (**e**) The plot of external quantum efficiency versus laser power intensity. (**f**) The time-resolved photoresponse spectra of p-GaN/SiO_2_/n-MoS_2_/graphene photo-detector measured under 633 nm laser illumination (laser power = 3.1 mWcm^−2^).

Figure 2a shows the 2D schematic structure consising of p-GaN/SiO_2_/n-MoS_2_/Graphene heterojunction for the measurement of photodetection. In this p-n heterojunction, the photogenerated carriers (electron-hole pair) can be originated from n-MoS_2_ and p-GaN, and then separated by the intrinsic built-in electric field at the interface, resulting in photo-diodes like characteristics as discussed below. Figure 2b displays the electrical characteristics (I–V curve) of the p-n heterojunction diode, showing an excellent rectification characteristics without light illumination, which provides an excellent evidence for the existence of the build-in electric field. Next, we investigate the photoresponse spectra of this fabricated p-n heterostructure device as shown in Fig. 2c, in which the topside of the 1-L MoS_2_ device is exposed to the light illumination. We perceived photoresponse when the visible light (λ = 633 nm; continuous wave laser) was used to illuminate the heterojunction with different intensities, and the induced photocurrent (I_ph_) was recorded. Under light illumination, the incident photons were mainly absorbed by n-MoS_2_ layer, and the generated electron-hole pairs were spatially seperated by internal built-in electric field. Interestingly, as seen from Fig. 2c under reverse bias, the current increases with increasing power density. The I_ph_ (I_ph_ = I_light_–I_dark_) was evaluated by deducting the current with and without light illumination. Note that 1-L MoS_2_ has a bandgap about 1.8 eV, which can absorb the photons of the excitation wavelength of 633 nm resulting in excellent photoexcitation of charge carriers. In addition, due to higher photocarrier extraction and collection efficiency arising from the inherent nature of single layer thickness, it leads to the high photocurrent. A more detailed discussion will be shown below.

Subsequently, we demonstrated the intensity-dependent I_ph_ of the heterostructure as a function of different illumination power density, as shown in Fig. 2d. Further, we calculated photoresponsivity (R_λ_) to examine the sensitivity of photodetection. R_λ_ can be defined as the *I*_*ph*_ generated per unit power of illuminated light and area, and described by the following equation, R_λ_ = I_ph_/(P_λ_S), where P_λ_ and S is the power of illuminated light and area of p-GaN/n-MoS_2_ heterojunction, respectively. The R_λ_ could reach ~10.4 AW^−1^ at 633 nm of 3.1 mW/cm^2^ illumination, which is the highest responsivity ever reported for 3D/2D p-n junctions. To present additional insight into the device performance, we measured wavelength dependent photo-diode characteristics as shown in Figure S2 in the supporting information, which is very close to the band gap absorption arising from 1-L MoS_2_. Table 1 summarizes the figures of merits of p-GaN/n-MoS_2_ heterostructure with other selected 3D-2D heterostructures for comparison. Obviously, our device has the best performance.

**Table 1:** Summary for the performance of photodetectors based on 3D-2D vertical stacking heterojunctions.

Heterojunction device type	Resposivity (A/W)	Detectivity	Response time	Ref.
n-Si/p-WS_2_	1.11	5.0 × 10^11^	42 ms	24
n-ZnO/p-MoS_2_	—	—	66 ms	28
GaN/hBN/MoS_2_	1.2 mA/W	—	500 ms	29
p-Si/n-MoS_2_	7.2	1.1 × 10^10^	—	44
p-GaN/SiO2/n-MoS2/graphene	10.4	1.1 × 1010	100 ms	Present work

To have a further insight into the photodetection performance, the external quantum efficiency (η) (EQE = hcR/eλ, where h, c, R, e and λ are the Planck’s constant, velocity of the light, photoresponsivity, the electron charge, and laser wavelength, respectively) of the heterojunction is calculated. Based on the illuminated power and photoresponse, η is evaluated by the equation: η = (I_ph_/e)/(P_in_/hν), where I_ph_, e, P_in_, h, and ν are the photocurrent, charge of electron, incident power, Planck’s constant, and frequency of light, respectively. Figure 2e shows the calculated η for our fabricated heterostructure. It is noted that an EQE as high as 30.00% was achieved, which is again the highest value ever reported for 3D-2D heterostructures. Furthermore, it is found that the η gradually decreases with increasing power density, which may be attributed to the screening of the internal field by the excited electrons and holes. One more important parameter to express the photoresponse characteristics is the specific detectivity (D*), which is determined by D* = R_λ_ S^1/2^/(2eI_dark_)^1/2^, where R_λ_, S, e, and I_dark_ are photoresponsivity, effective area, elementary charge, and dark current, respectively. From Figure S3, the D* is achieved approximately 1.1 × 10^10^ Jones, exhibiting that our heterojunction is a highly sensitive photodetector.

Furthermore, we have also examined the time-resolved photoresponse performance of our hererostructure photo-diode. The time dependent photocurrent (*I*_light_ − t) plot was carried out with a sequence of light irradiation by a 633 nm laser source. Figure 2f displays the *I*_light_ − t measurement at P = 3.1 mWcm^−2^, which exhibits a sharp increase of photocurrent when the light was on, and a sudden drop when it was turned off, indicating good stability and repeatability of our hererostructure. It is established that the rising and falling times were observed to be 0.25 s and 0.1 s, respectively. The consistent photo-switching behaviour with different cycles for the same device as shown in Figure S4 designates the stability and reproducibility of the device.

**Figure 3:**
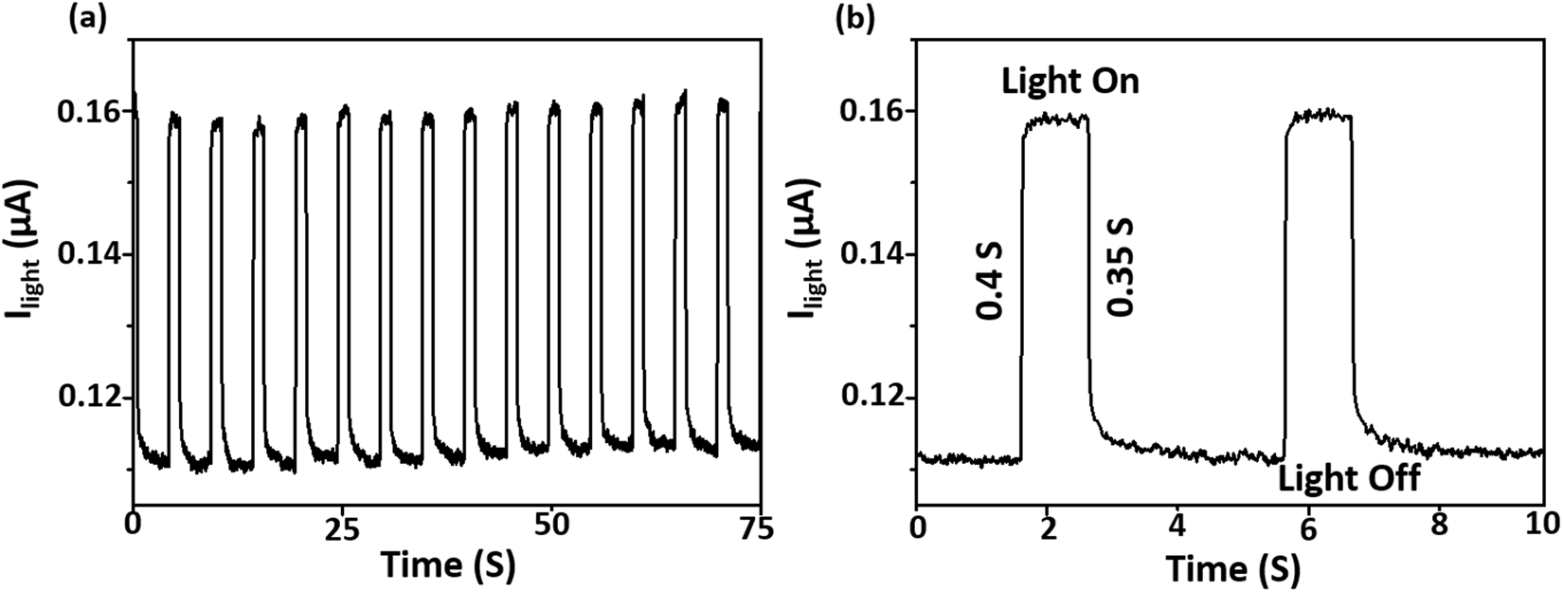
(**a**,**b**) Time-resolved photoresponse spectra of p-GaN/SiO_2_/n-MoS_2_/graphene self powered photo-detector measured at zero bias under 633 nm laser illumination (laser power = 3.1 mWcm^−2^).

Moreover, the effective separation of charge carriers due to the built-in electric field arising from p-n junction provides the capability of self-powered photodection for our device. Owing to the existence of the built-in electric field, the generated electrons and holes move towards opposite electrodes, leading to the generation of photocurrent without any external bias. Thus, the device can act as a self-powered photodetector. The photoresponse of the device at zero bias under illumination of 633 nm laser also exhibits an excellent photosensitivity and the steep rise and decay times of 0.4 and 0.35 as shown in Fig. 3, indicating that electron-hole pairs could be effectively generated and separated in the heterojunction under zero bias.

**Figure 4:**
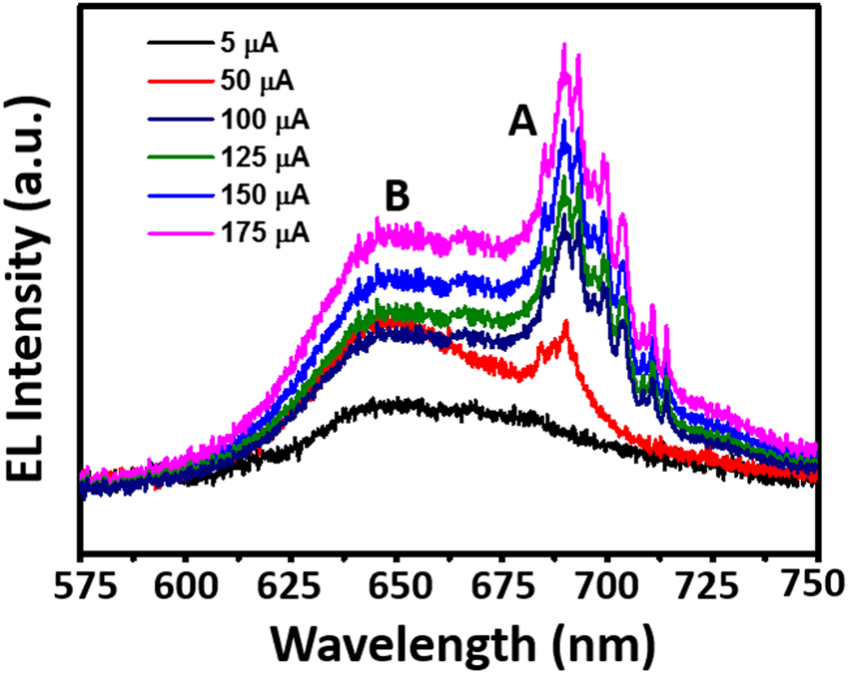
Electroluminescence from vertically stacked p-GaN/SiO_2_/n-MoS_2_/graphene heterostructure under different injection currents.

In addition to act as a photodetector, 3D-2D heterojunction diode also exhibits an obvious electroluminescence (EL) under forward bias as shown in Fig. 4. To realize efficient and broad area EL emission, a p–n junction (p-GaN/n-MoS_2_) is typically needed to stacked vertically. It is in sharp contrast with the lateral junction, which can only emit light at the local one-dimentional junction. However, vertically stacked electrically driven LEDs fabricated using atomically thin 2D materials could obstruct the carrier discharge for efficient accumulation of charge carriers^17^. This drawback can be overcome by the introduction of a thin insulating SiO_2_ layer. Upon applying an external electric field, the electrons are injected from graphene through the top electrode, simultaneously holes were injected from p-GaN through the bottom electrode. Near the thin SiO_2_ depletion layer, the injected electons and holes are accumulated and subsequently give rise to the efficient light emission. Intrestingly, clear excitonic A and B emissions from MoS_2_ were observed as shown in Fig. 4. Compared with the PL spectra shown in Fig. 3b, the EL peak position matches well with the PL peak, revealing the same exciton state. However, the EL spectra are much broader, which can be attributed to the electric field induced band bending^40^. Notably, the excitonic A emission becomes more pronounced as the injection current increases, which may be attributed to the self-absorption effect because the transition energy of B exciton is higher that of A exciton. In addition, the small oscillations in the EL spectra can be attributed to the effects of the superposition of both the interference patterns arising from the GaN substrate and defect states^17^.

**Figure 5:**
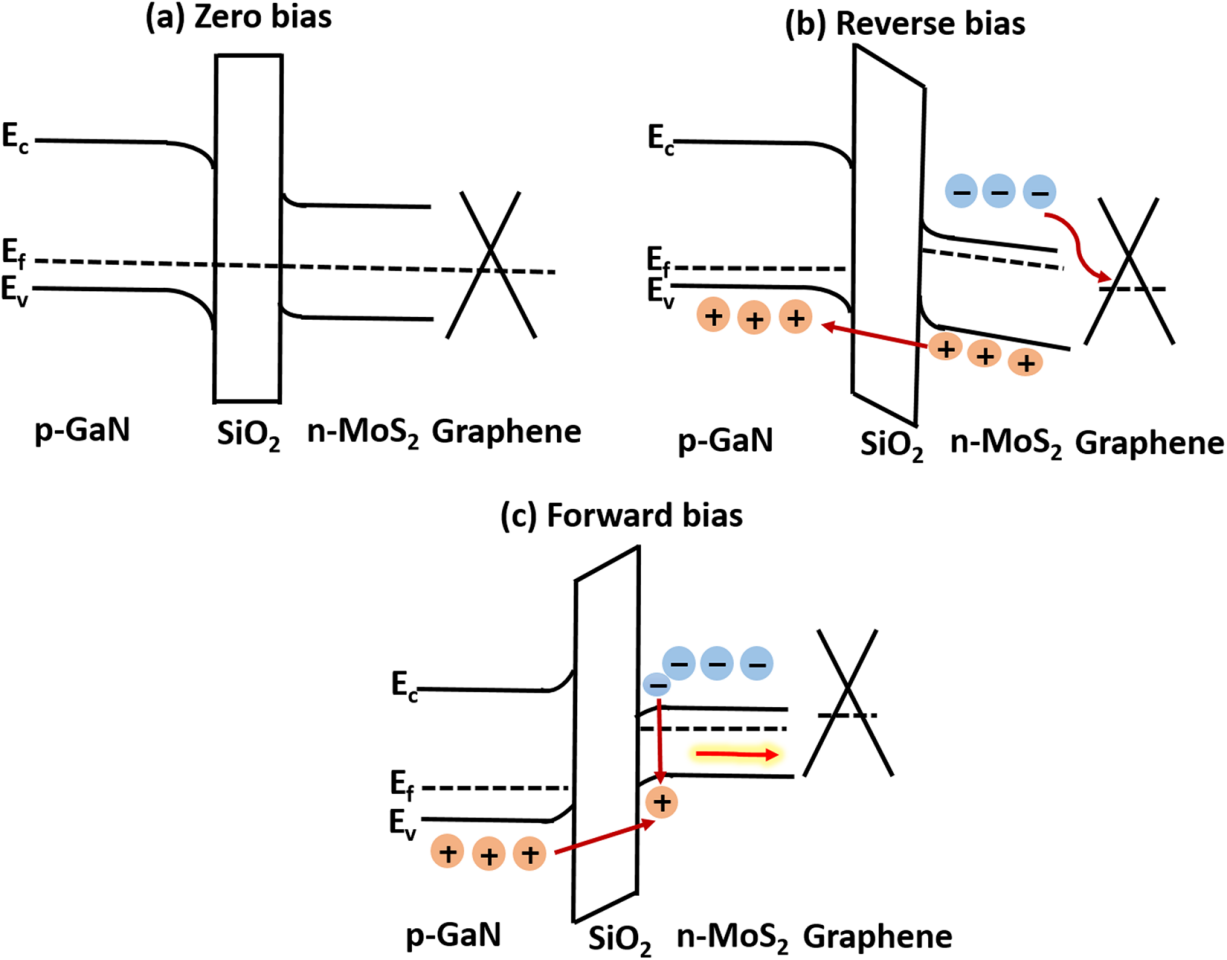
Energy band diagram of p-GaN/SiO_2_/n-MoS_2_/graphene heterojunction device under different bias condition. (**a**) At zero bias (V = 0), (**b**) Reverse bias condition (V < 0) and (**c**) Forward bias condition (V > 0).

To provide a more detailed understanding about the ascertained photodection and EL spectral characteristics under different bias conditions, we have scrutinized the energy band structure at the interface of the heterojunction. The underlying mechanisms are shown schemically in Fig. 5 and explained as follows. There are three important parameters (work function, electron affinity and bandgap of the materials) needed to draw the exact energy band diagram for a heterostructure device. The work function values for 1-L MoS_2_ and p-GaN are ~4.6–4.9 and ~7.5 eV, respectively^21, 41^ and the corresponding electron affinities are 4.2 and 4.1 eV, respectively^21, 42^. And the bandgap of 1-L MoS_2_ and p-GaN are 1.8 and 3.4 eV, respectively^21, 43, 44^. Figure 5a–c displays the enegy band alignment of the vertically stacked p-GaN/SiO_2_/1-L n-MoS_2_/Graphene heterojunctions. The SiO_2_ layer has a work function and bandgap about 5.4 eV and 6.97 eV, respectively, which can act as an insulating layer to eliminate leakage current and to accumulate injected carriers near the interface for carrier recombination and conduction depending on the polarity of applied bias. In order to have a better performance of the device, the thickness of SiO_2_ layer has to be optimized. For example, without the SiO_2_ layer, we can hardly detect light emission. And, if the insulator thickness is above 100 nm, the device performance will degrade drastically due to the strenuous tunnelling of charge carriers. Under negative bias (V < 0), the valence band of GaN will fall inside the bandgap of MoS_2_, whereas the the conduction band of MoS_2_ will fall inside the bandgap of GaN as shown in Fig. 5b. Therefore, upon light illumination, the photo-generated holes will tunnel across the thin SiO_2_ layer into GaN and electrons will transfer into the graphene layer. The highly sensitive photodetection of our device under reverse bias can thus be realized. Under forward bias, p-GaN can allow holes to tunnel from GaN to MoS_2_ and injected electrons will accumulate near the SiO_2_ interface. Consequently, the electrons and holes will recombine and generate emitted light efficiently.

Finally, in comparision with the most recent published work of double-heterojunction nanorod light-responsive LEDs^45^, which possesses the similar functionalities as our device shown here. It demonstrates that with the dual functions of photodetection and light emission, the devices can have a variety of potential applications, including touchless interactive panels and parallel data communication. However, the reported device was made by organic and inorganic hybrid materials with a complicate structure, which has a major drawback of reproducibility, reliability and durability unless the device is well encapsulated. This major difficulty can be overcome by using our device because it only consists of inorganic materials with a simple designed structure. In addition, due to the intensive efforts all over the world for two-dimensional materials, it is foreseeable that the practical applications of our device can be realized in the near future. For instance, it was found that the emission intensity of MoS_2_ based LEDs can be enhanced by more than 1000 times in multilayer MoS_2_ devices^40^.

## Conclusion

In summary, 3D-2D heterojunction diodes consisting of p-GaN/SiO_2_/n-MoS_2_/Graphene were fabricated and their excellent electrical and optical properties were demonstrated. Single layer MoS_2_ and graphene layers were assembled vertically by a wet chemical transfer technique on the p-GaN/SiO_2_ over the large area. From the electrical measurements, the p-n heterojunction diode revealed a good current-rectifying behaviour, resulting to the excellent light detection and emission characteristics. The observed high photoresponsivity and detectivity of the p-n heterojunction are ~10.4 A/W and 1.1 × 10^10^ Jones, respectively. These values are much better than most reported heterojunction devices. In addition, the photodetection of these heterojunction devices can be operated at zero external bias, thus reducing the external design and energy consumption. Moreover, the EL emission spectra in the range of ~680 nm from the vertically stacked heterostructures can be obtained based on a low turn-on voltage of 1 V. Our result provides an interesting platform for fundamental investigation of the carrier generation and recombination from 3D-2D heterostructure devices, and paves useful pathways to the integrated multifunctional optoelectronic devices, including photodetectors and light emitting diodes, which can have a wide variety of applications.

## Experimental Section

### Growth of monolayer MoS_2_

Monolayer MoS_2_ was grown using the home made three-zone tube furnace equipped with CVD reaction chamber at 700 °C. The MoO_3_ and sulfur powder are used as precursor to obtain n-type MoS_2_ on Si wafer containing a 300 nm thick SiO_2_ dielectric layer. Briefly, a Si/SiO_2_ substrate shielded with ~10 mg MoO_3_ powder was located in a small alumina crucible, which was placed at the middle of the furnace tube. The reaction chamber was evacuated by mechanical pump and then sealed properly. The MoO_3_ powders were heated gradually at ~25 °C/min to 700 °C for 30 min. At the same time, 0.5 g of sulfur powder, put down at the channel of the furnace which holds carrier-gas of Ar, was sublimated at ~170 °C and was circulated by 200 sccm Ar in the direction of the growth substrate retained at 700 °C. Then, the CVD chamber cooled slowly to the room temperature at the rate of 0.5°/min. The obtained film was approximately monolayer n-type MoS_2_, which was examined from colour interference perceived in the optical microscope and then it was determined by atomic force microscopy (AFM), Raman spectroscopy and elecrtical characteristics.

### Growth of monolayer grapheme

Graphene were grown by home-made chemical vapor deposition (CVD) technique on copper foil. The quality and thickness of the transferred graphene were verified by Raman spectroscopy (Jobin Yvon T64000).

### Device Fabrication

p-GaN (Mg doped GaN; doping levels of 9 × 10^17^ cm^−3^) on sapphire substrate was grown by metal-organic chemical vapor deposition technique. First, a p-GaN wafer was washed using acetone, isopropyl alcohol, and DI water, subsequently. To obtain a low resistance ohmic contact on p-GaN, Cr/Au was annealed after the deposition. A 10 nm insulating SiO_2_ film were deposited on p-GaN surface via RF sputtering after the emergence of Cr/Au ohmic contact. As-grown monolayer n-type MoS_2_ and graphene were transfered onto the p-GaN/SiO_2_ with the aid of polymethylmethacrylate (PMMA) and diluted HF, then the device was heated at 80 °C for 60 min to remove the residuals. The structure of the device was analysed using an optical microscope (Olympus, BX 51 M) equipped with a charge-coupled device (CCD) (Leica, DFC495).

### Characterization Details

All measurements were conducted in room temperature. An atomic force microscopy (AFM; Veeco D3000 NS49) was used to measure the thickness of the as-grown MoS_2_ layer on SiO_2_ substrate. The fabricated structure of the devices was confirmed by field emission scanning electron microscopy (FE-SEM; JEOL JSM6500). The electrical and optical characteristics (I–V curves) of the device were analyzed with the assistance of a conventional probe station (Lakeshore, TTPX) equipped with a power supply (Ophit, Nova II), source meter (Keithley, 2636 A) and an optical system, including a He-Ne laser (JDS Uniphase, Novette 1507), an optical beam shutter (Thorlabs, SH1), a Xenon lamp (Newport, 66921), and a monochromator (Acton, Spectrapro-500).

## Electronic supplementary material

Supplementary information
