# Supplementary material for: Diverse Functionalities of Vertically Stacked Graphene/Single layer n-MoS2/SiO2/p-GaN Heterostructures
Source: Sci Rep. 2017 Aug 30;7:10002. doi: 10.1038/s41598-017-09998-1 (PMC5577265; doi:10.1038/s41598-017-09998-1)
Supplement: Supplementary file 1 — Supplementary information [file 41598_2017_9998_MOESM1_ESM.pdf]

Supporting Information

Diverse Functionalities of Vertically Stacked Graphene/ Single layer n-MoS_2/SiO_2/p-GaN Heterostructures

Packiyaraj Perumal[1,2,3], Chelladurai Karuppiah[4], Wei-Cheng Liao[1], Yi-Rou Liou[1], Yu-Ming Liao[1] and Yang-Fang Chen[1,3]

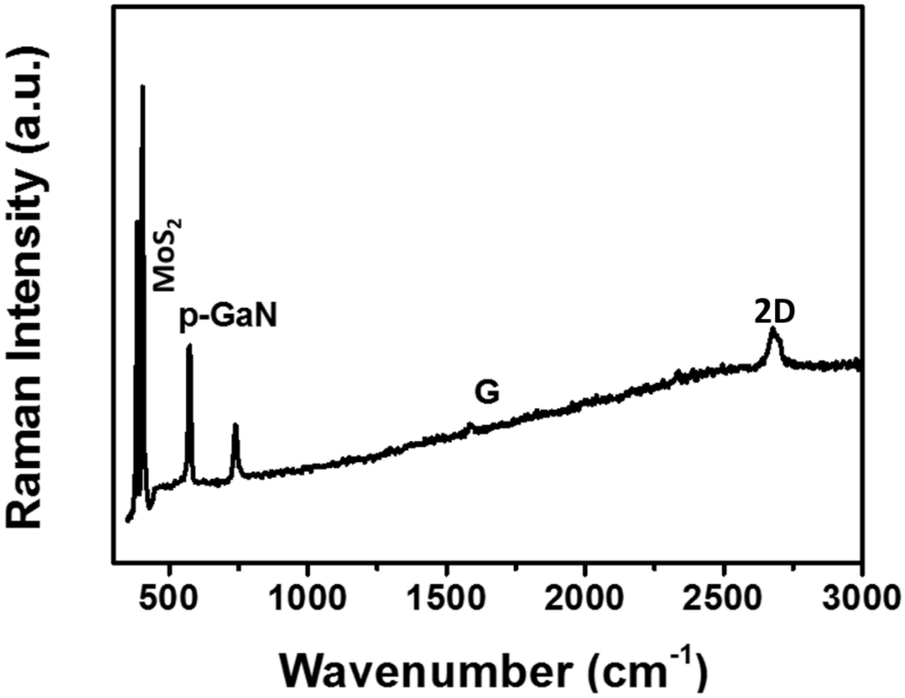

Figure S1. Confocal Raman spectra for vertically stacked p-GaN/SiO_2/n-MoS_2/graphene heterostructure.

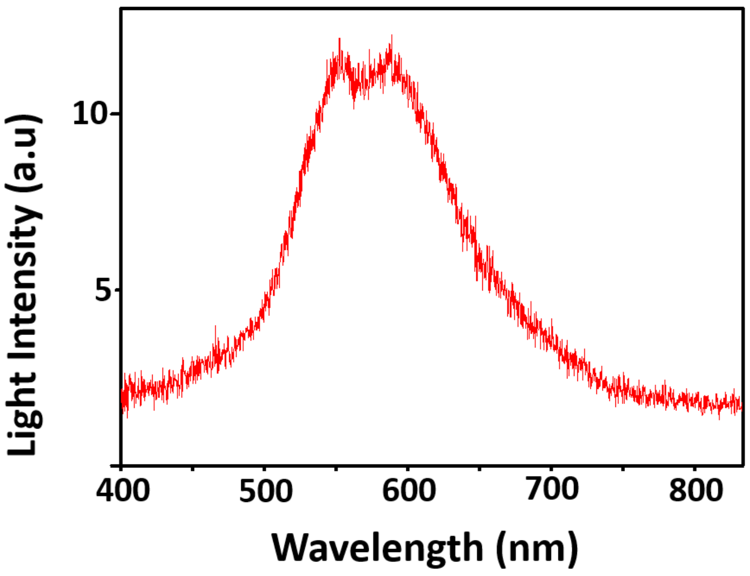

Figure S2. Wavelength dependent photocurrent measurement excited by visible light source.

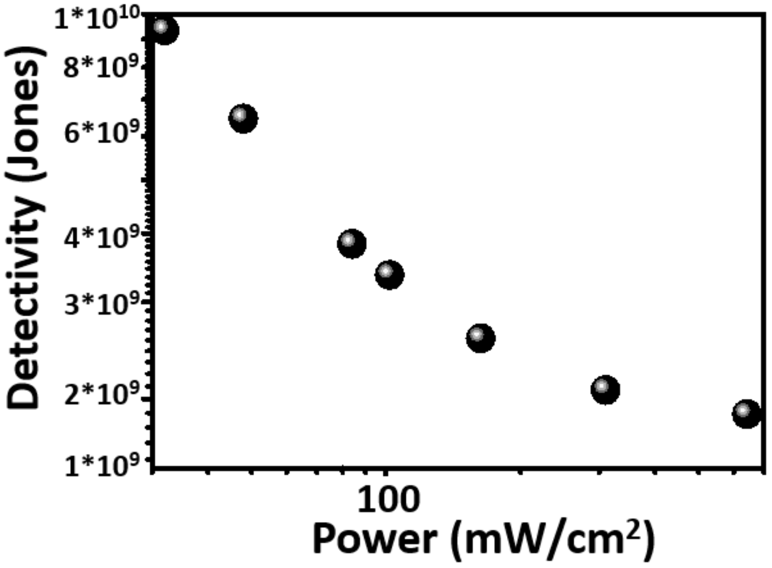

Figure S3. Plot of specific detectivity versus laser power intensity.

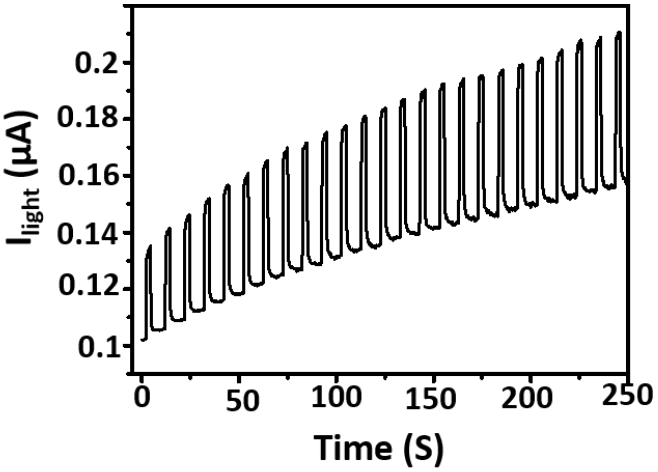

Figure S4. Time-resolved photoresponse spectra of p-GaN/SiO_2/n-MoS_2/graphene photo-detector measured under different cycles with 633 nm laser illumination (laser power = 3.1 $mWcm^{-2}$).
